# Activated biocarbons derived from molasses as new tailored CO_2_ adsorbents

**DOI:** 10.3389/fchem.2023.1184389

**Published:** 2023-06-19

**Authors:** Karolina Kiełbasa

**Affiliations:** Department of Catalytic and Sorbent Materials Engineering, Faculty of Chemical Technology and Engineering, West Pomeranian University of Technology in Szczecin, Szczecin, Poland

**Keywords:** activated biocarbon, molasses, CO_2_ adsorption, adsorption models, CO_2_/N_2_ selectivity

## Abstract

An innovative and cost-effective method for enhancing CO_2_ capture by modifying the textural properties of derived activated biocarbons was explored. A molasses solution was prepared with a sucrose concentration of 1 mol/dm^3^. A two-step synthesis process was involved, which includes the hydrothermal synthesis of spherical carbonaceous materials from molasses followed by chemical activation. The carbonaceous material to activation agent ratio was studied from 1 to 4. The CO_2_ adsorption of all activated biocarbons was tested at 0, 10, and 20°C and a pressure of up to 1 bar. The results showed a significant correlation between CO_2_ adsorption and the textural properties of the activated biocarbons. The activated biocarbon with the highest CO_2_ adsorption of 7.1 mmol/g at 1 bar and 0°C was successfully produced by modifying with KOH. The selectivity of CO_2_ over N_2_ calculated on the basis of the Ideal Adsorbed Solution Theory was excellent (16.5). The Sips model was found to be the most suitable, and the isosteric heats of adsorption were also specified.

## 1 Introduction

The global climate crisis and the growing commitment of industrial sectors to achieving net zero emissions by 2050 have prompted efforts to reduce greenhouse gas emissions, particularly CO_2_, which accounts for approximately 77% of all such emissions (Nyambura et al., 2011; Chen et al., 2022). As a result, significant attention is being given to the development of technologies aimed at reducing CO_2_ emissions, including Carbon Capture, Utilisation, and Storage (CCUS) (Aminu et al., 2017; Chiang and Pan, 2017). Despite the existence of several promising industrial methods at various stages of development, none of these technologies have yet proven to be economically viable and comprehensive enough for practical implementation.

Extensively investigations have been devoted to developing technologies, conducting to the capture and storage of CO_2_, particularly adsorption methods, which are nowadays very promising. Lots of adsorbents have been developed that could be used in the capture of carbon dioxide, e.g., carbonaceous materials (Srenscek-Nazzal et al., 2015; Mlodzik et al., 2016; Serafin et al., 2017) zeolites (Nguyen et al., 2016; Gesikiewicz-Puchalska et al., 2021) organometallic structures (Zhang et al., 2017) and porous polymers (Sun et al., 2015). Activated carbons have emerged as highly promising materials for CO_2_ sorption due to their ability to fulfill various requirements, such as chemical and thermal stability, low energy requirements for renewal, hydrophobicity, and stability during renewal (Ma et al., 2022). Moreover, when biomass or waste is used as the carbon precursor, these sorbents become an affordable option, and many biomass-based activated carbons exhibit both high adsorption capacity and selectivity (Sayari et al., 2011). In recent years, many studies have been focused on a very promising carbon spheres showing a full spectrum of applications (Hu et al., 2008; Wickramaratne and Jaroniec, 2013; Romero-Anaya et al., 2014; Boyjoo et al., 2017; Dassanayake and Jaroniec, 2018; Teague et al., 2019). Carbon spheres can be activated, for instance, with KOH, NaOH, ZnCl_2_, so they can be used as sorbents.

So far, many methods of obtaining carbon spheres have been developed. They were produced mainly by the Stöber method based on resorcinol reaction with formaldehyde in a water-alcohol-ammonia solution under conditions of hydrothermal synthesis at 100°C (Wickramaratne and Jaroniec, 2013). The spheres were then carbonized at 400°C–800°C under nitrogen. They activated under CO_2_ at 850°C. The obtained materials were characterized by a large specific surface area and a homogeneous structure. The highest CO_2_ adsorption under 1 bar pressure was, respectively: 8.05 mmol/g and 4.40 mmol/g at 0°C and 25°C. Marszewska and Jaroniec modified the Stöber method by adding tetraethoxysilane or colloidal silica to the reaction mixture, thanks to which they obtained carbon nanospheres, from which the templates were removed at 60°C using a KOH solution at a concentration of 3 mol/dm^3^ (Marszewska and Jaroniec, 2017). Modification of the Stöber method by adding K_2_C_2_O_4_·H_2_O made it possible to eliminate the activation step and obtain CO_2_ adsorption of 6.6 mmol/g at 0°C (Ludwinowicz and Jaroniec, 2015). The carbon spheres obtained by the Stöber method were also activated by stirring for 2 h in KOH solutions of various concentrations. Then they were washed and carbonized (Wang et al., 2017).

At 0°C, the CO_2_ adsorption was 7.34 mmol/g, while at 25°C, it was 4.83 mmol/g. Dassanayake and Jaroniec also created carbon spheres using 3-aminophenol and pyrrole, as well as ammonium persulfate and p-toluenesulfonic acid (Dassanayake and Jaroniec, 2018). The spheres were calcined in a nitrogen atmosphere at 600°C and then activated with KOH at 600°C and 700°C. The maximum carbon dioxide adsorption at 1 bar was 7.73 mmol/g and 5.42 mmol/g at 0°C and 25°C, respectively. Additionally, sweet drinks such as Coca Cola and Push Orange were subjected to autoclaving at 220°C for 24 h, and the resulting material was carbonized in a nitrogen stream at 1,000°C. At 1 bar, the CO_2_ adsorption was 4.65 mmol/g and 2.99 mmol/g at 0°C and 25°C, respectively (Teague et al., 2019).

Various publications have discussed the preparation of carbon spheres using chemical substrates like resorcinol and formaldehyde. However, an alternative approach for obtaining carbon materials involves using waste biomass from the food industry, specifically molasses, and KOH as an activator. This method offers an environmentally-friendly solution for producing carbon spheres without relying on traditional chemical raw materials.

In this study, the characteristics of activated carbons were regulated by various factors including hydrothermal synthesis parameters, carbonization temperature, chemical activation, and the ratio of carbonaceous materials to activating agent. The impact of the controlled textural parameters of the activated biocarbons on their ability to adsorb CO_2_ was examined.

As far as it knows, there are no prior publications regarding the two-step synthesis process used in this study, which involves the hydrothermal synthesis of spherical carbonaceous materials from molasses (a waste product of the sugar industry) followed by chemical activation. This method avoids using biomass that could impact food supplies, making it an attractive option. These findings demonstrate that cost-effective activated biocarbons can be generated from molasses as the starting material with excellent efficiency.

Moreover, it was shown for the first time that as a result of mild activation of carbon spheres which were hydrothermal synthesized, porous materials, CO_2_ sorbents can be obtained without destroying the spherical structures. Authors who used a similar method of synthesis described the destruction of spherical structures or did not present the morphology of materials after activation.

## 2 Methods and materials

The raw material for the preparation of carbon materials was beet molasses—waste from the sugar industry containing approximately 50% of sucrose. A molasses solution was prepared with a sucrose concentration of 1 mol/dm^3^. The solution was placed in an autoclave for 12 h at the temperature of 200°C. After the completion of the hydrothermal synthesis, the sample was removed from the autoclave, rinsed with deionized water until it was neutral pH, and then dried at 110°C. The sample was labeled as MB.

After drying, the material obtained from a hydrothermal synthesis with 1 mol/dm^3^ sucrose solution was modified with KOH for 3 h. The mass ratio of carbon to activator was Chen ed in the range from 1 to 4. The samples were denoted as MB_1:1, MB_1:2, and MB_1:4, respectively. The material was heated at the temperature of 750°C. The combined carbonization and the activation procedure was conducted under a nitrogen atmosphere (flow 20 dm^3^/h). The activated carbons with the decomposition products of potassium hydroxide were thoroughly washed with deionized water until the solution became neutral. Then, the sample of carbonaceous material was flooded with HCl solution with a concentration of 1 mol/dm^3^ and left for 20 h. Carbonaceous material was rinsed with deionized water until it reached a neutral pH. In the final stage, the material was dried at 110°C for 20 h to obtain the desired biocarbon.

The structure of the received biocarbon was analyzed using the Hitachi SU 8200 field emission scanning electron microscope.

The phase composition of the biocarbons was analyzed using a PANalytical Empyrean X-ray diffractometer (XRD) equipped with a Cu anode to generate Kα radiation. The diffraction patterns obtained were compared with standard diffraction patterns from the ICDD PDF4+2015 database using X’Pert HighScore computer software to identify the location and intensity of reflections.

Raman spectroscopy was employed to determine the structure of the prepared biocarbons. The analysis was performed using a Renishaw InVia apparatus equipped with a CCD detector. A laser with a wavelength of 785 nm was used to induce the samples, and the spectrum was obtained in the range of Raman scattering from 800 cm^−1^ to 2000 cm^−1^. After normalizing the G peak maximum to 1, the intensity and location of the G and D peaks were identified, and the ratio of these intensities was calculated. The G and D band intensity ratio is widely recognized in the literature as a method for determining the order of graphene layers and graphitic structure in carbon materials.

The carbon materials were characterized texturally using a Sorption Surface Area and Pore Size Analyzer (ASAP 2460, Micrometrics, Novcross, GA, United States), and the control and data acquisition were enabled by the ASAP software. To remove pollutants before the measurements, the carbon samples were calcined at 250°C for 12 h under reduced pressure with a heating rate of 1°C/min. Low-temperature N_2_ adsorption isotherms were measured at −196°C, and specific surface area (S_BET_), total pore volume (V_p_), micropore volume (V_mi_) determined by the DFT method (the Density Functional Theory), and mesopores volume (V_ms_) determined by the BJH method (Barrett, Joyner and Halenda method) were obtained based on the measurements of N_2_ adsorption.

The adsorption studies of CO_2_ were performed at pressures up to 1 bar and temperatures of 0°C, 10°C, and 20°C using the ASAP apparatus. To control the temperature during measurements, the sample was placed in a water bath with a Peltier-cooled solid-state detector. Prior to the CO_2_ adsorption measurements, the tested materials were outgassed at 250°C for 12 h.

## 3 Results and discussion

**FIGURE 1:**
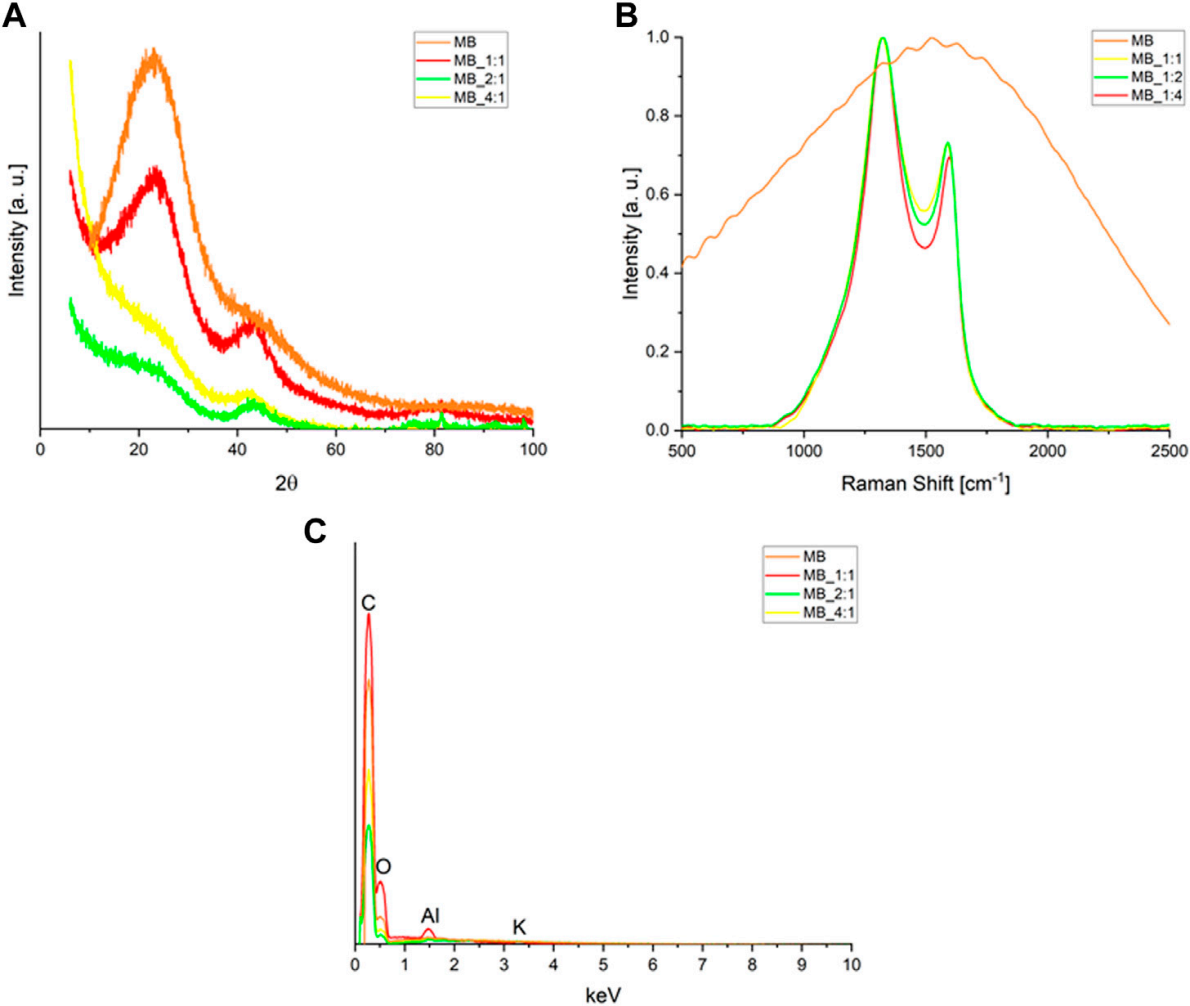
**(A)** XRD diffraction patterns, and **(B)** Raman spectra of the activated carbons produced from molasses after the smoothing, baseline subtracting, and normalizing to the D band intensity **(C)** the Energy-dispersive X-Ray Spectroscopy (EDS) spectrum.

XRD was utilized to characterize the graphitic structure and purity of the activated biocarbons. The obtained diffraction patterns of the activated biocarbons are presented in Figure 1A. Two broad asymmetric peaks were observed in the diffraction pattern of activated carbon MB_1:1 at 2θ angles of approximately 23° and 43°. These correspond to the planes (002) and (100/101) of the graphite structure (JCPDS 41-1487) associated with the stacking height-thickness of the layer packets (Lc) and longitudinal dimension, respectively. The broad peaks indicate a highly disordered carbon structure and a mostly amorphous arrangement.

Clear broad peaks at 2θ about 23° disappear for biocarbons with a higher ratio of activator, i.e., MB_1:2 as well as MB_1:4, moreover, their XRD spectra contained low-intense signals. Such a high disorder is usually associated with high values of specific surface area, total pore volume, and micropore volumes. All these parameters play a significant role in CO_2_ adsorption. On the other hand, analyzing the XRD spectra, it was observed that the materials obtained after hydrothermal synthesis and using the lowest ratio of carbon material to KOH were characterized by relatively high intensities. This proves a relatively higher degree of ordering of these materials. It is possible to hypothesize that the reaction of carbon with KOH occurs to a lesser extent under mild conditions, such as low activator content.

The degree of ordering of the produced carbon materials was assessed using Eqs 1–6:

$$L=\frac{K\lambda_{XRD}}{\beta \cos\left(\theta_{hkl}\right)}$$ (1)

where L is the mean crystallite dimension in nm along a line normal to the reflecting plane; λ_XRD_ is the X-ray wavelength (for copper K_α_ radiation λ_XRD_ is 0.15409 nm); K is a constant depending on the reflection plane; β is the full width at half-maximum of the (100/101) and (002) peaks in radians of 2θ after subtraction of the instrumental broadening; θ_hkl_ is the scattering angle (in radians).

The instrumental broadening effect on full width at half-maximum was subtracted out using Warren’s method assuming a Gaussian peak (Cullity and Stock, 1978):

$$\beta =\sqrt{\beta_{meas}^{2}-\beta_{inst}^{2}}$$ (2)

Eqs 3, 4 were used to calculate L_a_ and L_c_ based on (100/101) and (002) data, respectively. The constant K was set to 0.89 for L_c_ and 1.84 for L_a_ (Lu et al., 2001).

$$L_{a}=\frac{1.84\lambda_{XRD}}{\beta_{100/101}\cos\left(\theta_{100/101}\right)}$$ (3)

$$L_{c}=\frac{0.89\lambda_{XRD}}{\beta_{002}\cos\left(\theta_{002}\right)}$$ (4)

The average spacing between graphitic layers was calculated using the Bragg law (5):

$$d_{002}=\frac{n\lambda_{XRD}}{2\sin\left(\theta_{002}\right)}$$ (5)

Where n is an integer, it is commonly accepted in the literature that the order of reflection is typically considered to be 1.

The number of graphitic layers (N) was estimated using the equation:

$$N=\frac{L_{c}}{d_{002}}$$ (6)

However, it should be mentioned that 1st equation was derived for carbons with a high degree of graphitization and does not allow us to determine the average crystallite thickness (L_c_) and the average diameter of a graphene sheet (L_a_) with high accuracy for highly disordered carbons. However, they can be used for carbons with a disordered structure for a rough estimation of the values characterizing crystallites and for conducting a comparative analysis (Girgis et al., 2007). In fact, the crystallite sizes are probably slightly higher than those calculated on the basis of formulas (1) - (3). The values of average spacing between graphene layers, the diameters of graphene sheets, the thickness of crystallites, and the number of graphene layers are presented in the tables.

Table 1 presents the structural parameters obtained from XRD measurements. The activated carbon MB_1:1 exhibited the lowest average spacing between graphitic layers (0.368 nm), while the highest spacing (0.399 nm) was observed for activated carbon MB_1:4, which had the highest ratio of carbon material to KOH. The interlayer spacings of the obtained carbon materials are higher than that of graphite (0.335 nm), possibly due to sp3 defects and/or interlayer repulsion between surfaces with negatively charged functional groups (Ghosh et al., 2019).

**TABLE 1:** Structural parameters of activated carbons obtained from XRD, and I_G_/I_D_ ratios calculated from Raman measurements.

Carbon material	d_(002)_ [nm]	La [nm]	Lc [nm]	N	I_G_/I_D_
MB	0.395	2.013	0.865	2.190	
MB_1:1	0.368	2.958	0.735	1.999	0.733
MB_1:2	0.377	3.053	0.798	2.120	0.725
MB_1:4	0.399	3.333	0.989	2.481	0.695

The number of graphitic layers in the packets for all samples is assumed to be two. The dimensions of the aromatic sheets (L_a_) increased with the activation agent ratio and ranged from 2.013 to 3.333 nm.

**FIGURE 2:**
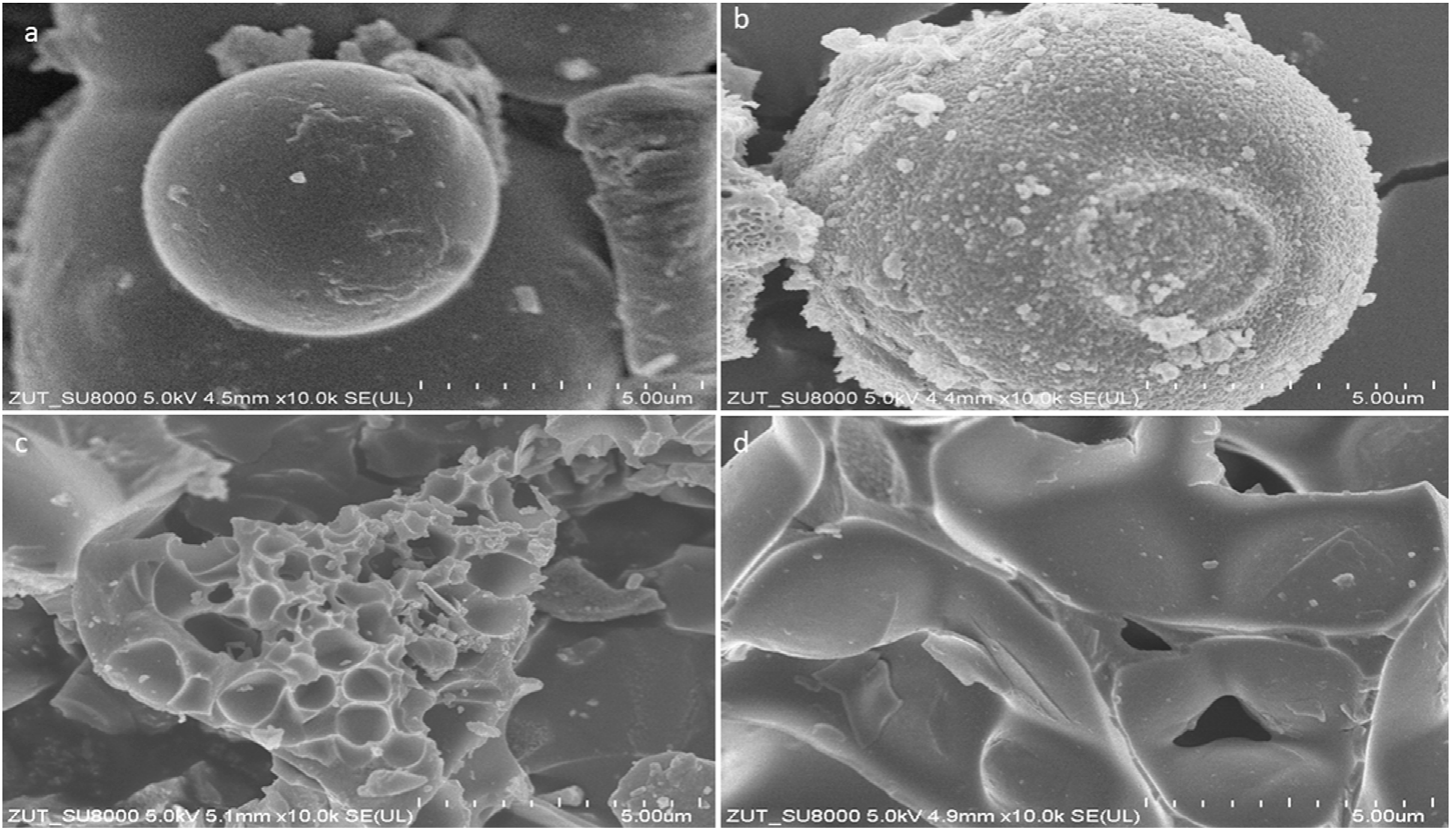
SEM pictures of the carbon materials: **(A)** MB **(B)** MB_1:1 **(C)** MB_1:2 **(D)** MB_1:4.

Raman spectroscopy is frequently utilized to assess carbon’s crystallographic defects and disorders. Figure 1B displays Raman spectra within a range of Raman shifts from 500 to 2,500 cm^−1^ for the activated materials. Two broad overlapping peaks were observed, the first one centered near 1,330 cm^−1^ representing the disordered portion of the carbon, and the second one centered near 1,600 cm^−1^ representing ordered graphitic crystallites of the carbon (sp2 bonding carbon atoms) –G band. The intensities of the D signals were higher than the G ones. The D and G band intensities were normalized, and the values of the G peak maxima in Figure 1B were equivalent to the I_G_/I_D_ intensity ratios. The ratios of the G and D bands were compiled in Table 1. The I_G_/I_D_ ratio can be utilized to estimate the degree of defects, where lower values indicate more defects. The presence of the G band in the Raman spectra implies the presence of graphene sheets. The lower the intensity ratio, the higher the disorder of the graphene sheets. The I_G_/I_D_ ratios for activated carbon samples ranged from 0.695 to 0.733, and the smallest values were found for MB_1:4. The absence of G and D bands in the MB sample indicated that it had a significant number of defective carbon structures. The results from XRD were consistent with those from Raman spectroscopy. The EDS spectrum (Energy-dispersive X-Ray Spectroscopy) of obtained carbonaceous materials shows only peaks for carbon, oxygen, aluminum, and potassium (Figure 1C). The surface elemental composition estimated by the EDS method of carbons materials was compiled in Table 2. Peaks for oxygen and potassium appear only for sample hydrothermally synthesized (MB), for other samples disappeared. It confirmed that samples activated by KOH were perfectly rinsed and do not contain any products of KOH decomposition. Aluminium probably is connected with aluminum from SEM (Scanning Electron Microscope) tables. The textures of the obtained materials were shown in SEM pictures (Figure 2).

**TABLE 2:** The surface elemental composition estimated by EDS method of carbons materials.

Carbon material	C [wt%]	Error	O [wt%]	Error	Al [wt%]	Error	K [wt%]	Error
MB	59.302	±0.305	39.556	±0.626	0.876	±0.027	0.266	±0.017
MB_1:1	99.512	±0.523			0.488	±0.092		
MB_1:2	99.266	±0.562			0.734	±0.110		
MB_1:4	99.199	±0.563			0.801	±0.119		

It is seen that sample after hydrothermal synthesis had a spherical structure. The grains are spherical and slightly deformed, and their diameters range from 6–10 μm. The morphology of the biocarbons activated under mild conditions, by the smallest carbon material: KOH ratio, looked similar, the sample had a spherical structure, however, spheres are more aggregated and deformed and porous surface. The appearance of activated biocarbon treated with a higher concentration of KOH differed noticeably, resembling petals with a rippled texture and uneven edges. In contrast, activated biocarbon samples MB_1:2 and MB_1:4 exhibited a more solid, undulating surface. Zhao et al. (2017) have proposed an explanation of the spherical shape of material derived from sucrose. It can be assumed, that samples derived from molasses, which was used as a precursor for described biochars, contain approximately 50% of sucrose, and exhibited similar behaviour. Namely, during hydrothermal conditions sucrose hydrolysed, resulting to the production of glucose and fructose. Subsequently, glucose, fructose, and other decomposition products present in the solution go through intermolecular dehydration and aldol condensation reactions, leading to polymerization. These polymers subsequently undergo aromatization, resulting in the creation of aromatic compounds. The formation of aromatic clusters then takes place through the intermolecular dehydration of these aromatic compounds. When the aqueous solution reaches a state of super-saturation, aromatic clusters will aggregate to form a central core. This nucleation process follows the model proposed by LaMer (Jain et al., 2016), resulting in the formation of hydrochar spheres. Subsequently, through heat treatment in an N_2_ atmosphere, the hydrochar spheres undergo the removal of small organic molecules. This removal process generates porosity within the spheres, ultimately leading to the formation of the final microspheres of activated carbon. It is important to note that the hydrochar serves as a crucial super-polymer with a specific carbon skeleton during this process, as it plays a critical role in the development of porosity. Taking into account, that in these preliminary studies, it was evidenced, that the ratio of additives of activator influences on the shape and porosity of derived materials, it has to be comprehensively examined in the future what is the exact nature of their roles.

**FIGURE 3:**
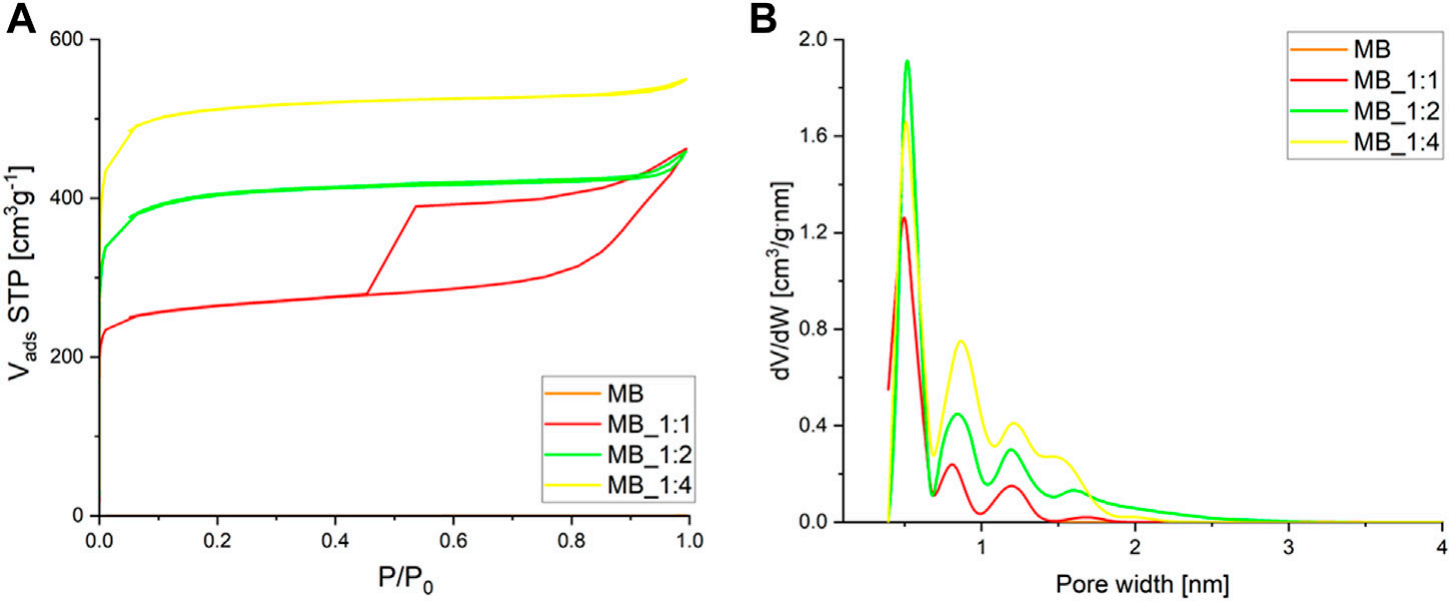
**(A)** Adsorption–desorption isotherms of nitrogen **(B)** and micropore pore size distribution calculated by the DFT method.

The nitrogen adsorption isotherms at −196°C are shown in Figure 3A.

The adsorption isotherms of all samples exhibited rapid growth at low pressure P/P_0_, indicating their microporous nature, as they were derived from spherical carbonaceous materials synthesized through hydrothermal processes. Hysteresis loops were observed for MB_1:1, indicating the presence of mesopores in addition to micropores, with the ratio of activation agents contributing significantly to their formation. The hysteresis loops for two samples MB_1:2 and MB_1:4 were very narrow, even invisible without proper magnification. MB_1:4 had the highest nitrogen adsorption rate and was classified as Type Ib according to IUPAC (International Union of Pure and Applied Chemistry), while MB_1:2 and MB_1:1 were a combination of Types Ia and IV. The isotherms were reversible, with some showing hysteresis caused by capillary condensation in mesopores, identified as H4 type for MB_1:1 that can be correlated to narrow slit-like pores.

The micropore size distribution, determined using the DFT method, showed dominant pores of about 0.5 nm in diameter, with MB_1:2 having the highest pore volume of such size. Table 3 summarizes the textural properties of the samples, with MB_1:4 having the highest surface area, total pore volume, and micropore volume, while MB_1:1 had the highest mesopore volume determined by the BJH method. Detailed analysis of macropores was unnecessary as they are not critical for CO_2_ adsorption and primarily serve to transport adsorbents to micro and mesopores. In this study, the maximum specific surface area was 2005 m^2^/g for biocarbon activated by the highest ratio of KOH: carbon. Compared to the literature data it shows quite good properties. For instance, carbon material synthesized from sucrose after hydrothermal conditions achieved a specific surface area equal to 1,180 m^2^/g after activation with CaCl_2_, and 1,529 m^2^/g after activation with H_3_PO_4_ (Zhao et al., 2017). Another example of carbon materials derived from hydrothermal treatment of sucrose exhibited a specific surface area equal to 3.06 m^2^/g without chemical activation (Hao et al., 2016). After carbonization, and activation with KOH surface area of derived activated carbons increases up to 2,837 m^2^/g. Ahmed et al. (Ahmed et al., 2017) as biomass precursor used bamboo, which was thermal treated (N_2_) at 380°C, and followed by activation with H_3_PO_4_ at 600°C. That adsorbent achieved a specific surface area equal to only 1.12 m^2^/g. Other researchers, which used hydrothermal conditions, prepared carbon materials from, e.g., grape speeds (hydrothermal treatment at 220°C, activation by KOH at 750°C) exhibited specific surface area up to 2,194 m^2^/g (Diaz, et al., 2022) or from dewatered waste activated sludge (hydrothermal carbonization at 208°C, and activation with KOH at 850°C) has a specific surface area equal to 832 m^2^/g (Villamil et al., 2020).

**TABLE 3:** Textural properties of carbons materials.

Carbon material	S_BET_ [m^2^/g]	V_p_ [cm^3^/g]	V_mi_ [cm^3^/g]	V_ms_ [cm^3^/g]
MB	0.3			
MB_1:1	1026	0.710	0.322	0.422
MB_1:2	1527	0.715	0.526	0.091
MB_1:4	2005	0.851	0.682	0.077

The findings from Figure 3 were consistent with the values obtained for textural properties, as shown in Table 3.

The data presented in Tables 1, 3 revealed that the pore volume was affected by the ratio of activator used during the production process. Activated carbons prepared using a lower ratio of KOH had a more ordered structure, resulting in lower porosity compared to those prepared with a higher ratio of KOH. This was attributed to the disordered structure of the latter, which provided the highest porosity.

**FIGURE 4:**
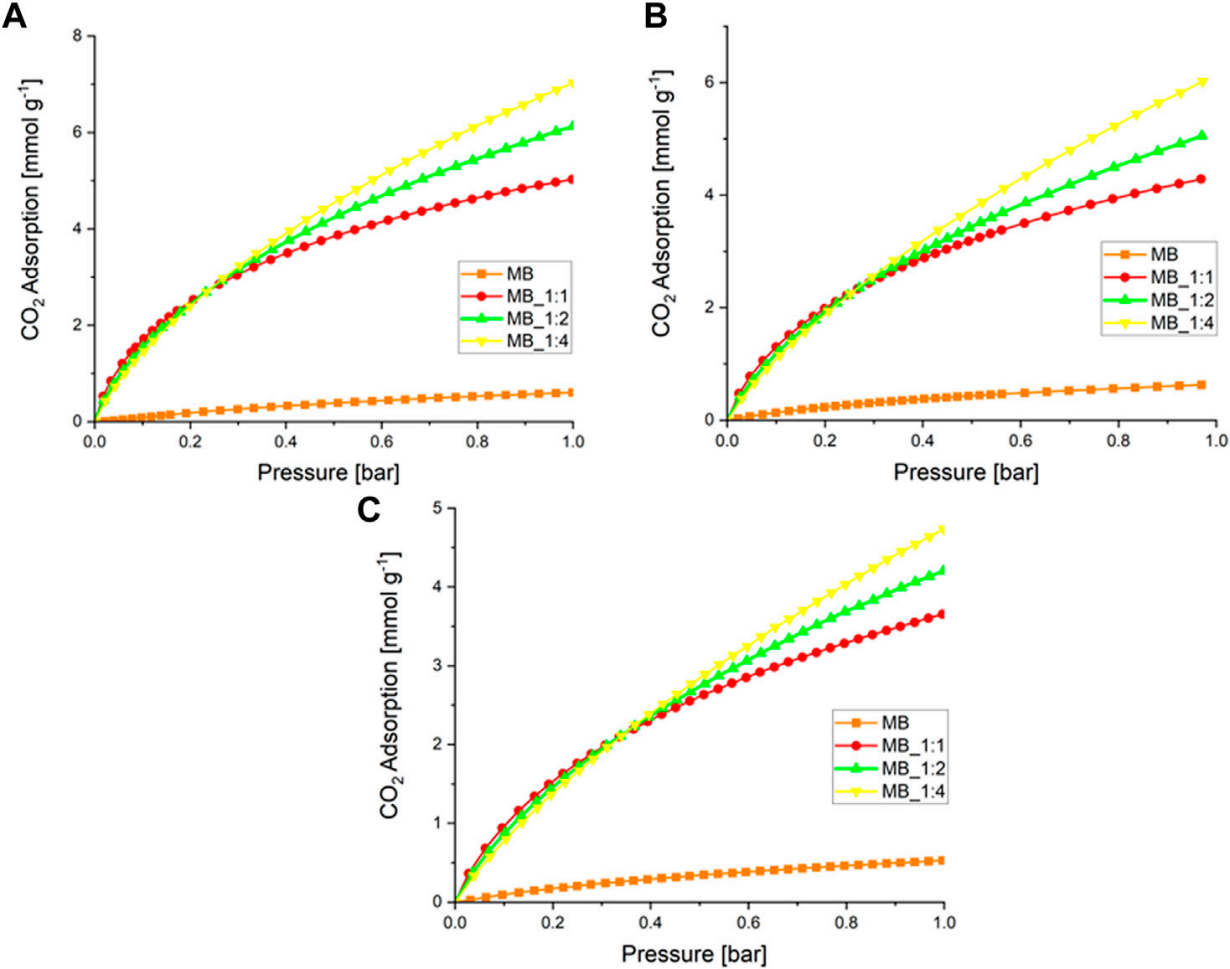
**(A)** CO_2_ adsorption at a temperature of 0°C **(B)** 10°C, and **(C)** 20°C over activated biocarbons.

The adsorption of CO_2_ was evaluated by subjecting the samples to pressures of up to 1 bar at temperatures of 0°C, 10°C, and 20°C. The adsorption results are shown in Figure 4.

Table 4 presents the results of CO_2_ adsorption at a pressure of 1 bar and various temperatures.

**TABLE 4:** The CO_2_ adsorption.

	The CO_2_ adsorption [mmol/g]		
	Temperature [°C]		
Carbon material	0	10	20
MB	0.63	0.60	0.53
MB_1:1	5.03	4.28	3.65
MB_1:2	6.13	5.05	4.20
MB_1:4	7.10	6.02	4.73

The activated carbons that were produced with the highest amount of KOH showed the highest CO_2_ adsorption, while the lowest values were observed for the sample produced through hydrothermal synthesis. Previous studies have suggested that CO_2_ adsorption is related to textural properties, particularly micropore volume (Serafin et al., 2017; Serafin et al., 2019; Serafin et al., 2022), which was confirmed by our results except for MB. The highest CO_2_ adsorption at 0°C and 1 bar was 7.1 mmol/g (MB_1:4), which is relatively high compared to other studies that claimed high CO_2_ adsorption performance, such as 3.31 mmol/g at 0°C and 1 bar (Yuan et al., 2022), and 3.2–5.3 mmol/g at 0°C and 1 bar (Spessato et al., 2022). The detailed comparison with the literature data already published was compiled in Table 5. The CO_2_ adsorption decreased as the temperature increased, indicating that CO_2_ sorption on activated carbon is mainly physical, regardless of the activating agent.

**TABLE 5:** The CO_2_ adsorption on various carbonaceous materials.

Carbon material	Preparation methods	Adsorption conditions	The CO_2_ adsorption [mmol/g]	Ref
Commercial carbon WG12	Activation with KOH	40°C, 1 bar	2.1	Srenscek-Nazzal et al. (2015)
Commercial carbon WG12	Activation with ZnCl_2_	40°C, 1 bar	1.6	Srenscek-Nazzal et al. (2015)
Waste wool	Carbonization-activation with KOH	0°C, 1 bar	3.7	Li et al. (2018)
Glucose	Hydrothermal carbonization-activation with KOH	0°C, 1 bar	4.9	Martin-Jimeno et al. (2015)
Biochar from cornstalks	Carbonization-activation with K_2_CO_3_	0°C, 1 bar	3.3	Yuan et al. (2022)
Local coals	Activation with NaOH	0°C, 1 bar	9.1	Toprak and Kopac (2017)
Biochar from Brazil nut shells	Carbonization-activation with KOH	0°C, 1 bar	5.3	Spessato et al. (2022)
Activated carbon from raw molasses	Carbonization-activation with KOH	0°C, 1 bar	5.4	Kielbasa et al. (2021)
Petroleum coke	Carbonization-activation with KOH	25°C, 1 bar	3.5	Hu et al. (2011)
Biochar from almond shell	Carbonization-activation with CO_2_	25°C, 1 bar	2.6	Plaza et al. (2010)
Biochar from soybean	Carbonization-activation with ZnCl_2_/CO_2_	30°C, 0.15 bar	0.93	Thote et al. (2010)
Carbon spheres from resorcinol	Hydrothermal treatment, carbonization-activation with CO_2_	0°C, 1 bar	8.05	Wickramaratne and Jaroniec (2013)
Carbon spheres from resorcinol	Hydrothermal treatment, carbonization-activation with KOH	0°C, 1 bar	7.34	Dassanayake and Jaroniec (2018)

The CO_2_ adsorption data for all activated sorbents at different temperatures and a pressure of 1 bar were presented in Table 4. The Langmuir, Freundlich, Langmuir-Freundlich (Sips), Toth, Fritz-Schlunder, and Radke-Prausnitz equations were used to model the experimental data. The equations that define the absolute amount of adsorbed gas as a function of pressure were presented in the Supplementary Material (Supplementary Equations S1–S9), and the sum of the squares of errors (SSE) was used as an error function (Supplementary Equation S10). The Sips model provided the best fit for the experimental data.

The calculated values of constants q_mS_, b_S_, and n_S_ in the Sips Supplementary Equation S3 for MB_1:1, MB_1:2, and MB_1:4 at different temperatures were presented in Supplementary Table S1. These parameters depend on temperature according to Supplementary Equations S4–S6, and plots of ln(q_mS_) versus T, ln(b_S_) versus 1/T, and n_S_ versus 1/T were created (Supplementary Figures S1–S3). Using Supplementary Equations S4–S6 and Supplementary Table S2, the parameters q_m0_, χ, Q, b_0_, n_0_, and *a* were estimated and presented in Supplementary Table S2. With this information, the CO_2_ adsorption over MB_1:1, MB_1:2, and MB_1:4 can be calculated at any temperature and pressure.

The calculation of the isosteric heat of adsorption, which represents the change in enthalpy at a constant coverage (θ), was performed using the Clausius-Clapeyron Eq 7 and then transformed into linear form (8).

The isosteric heat of adsorption:

$$E_{iso}=-R{\left(\frac{\partial \ln\left(p\right)}{\partial \left(\frac{1}{T}\right)}\right)}_{\theta }$$ (7)

The equation can be expressed in a linear form as follows:

$${\ln\left(p\right)}_{\theta }=-\frac{E_{iso}}{R}\frac{1}{T}+C$$ (8)

**FIGURE 5:**
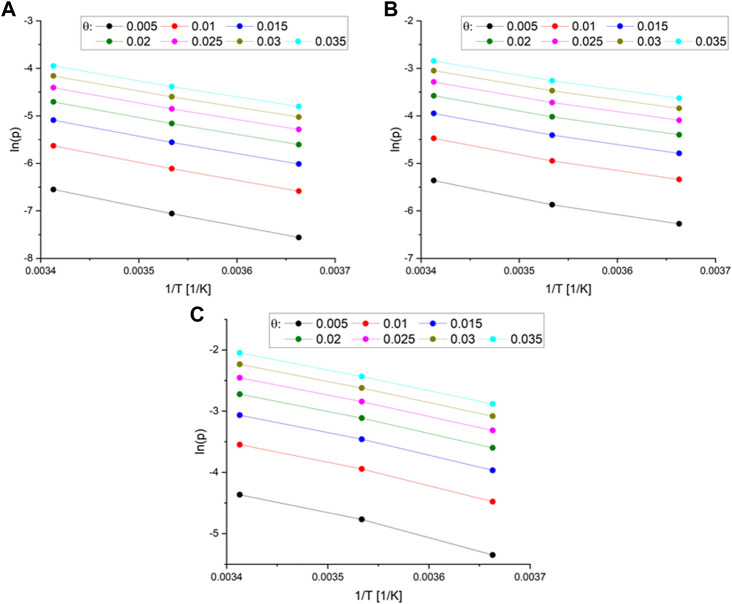
**(A)** The plot of the function ln(p) vs. 1/T different surface coverage for MB_1:1 **(B)** MB_1:2 **(C)** and MB_1:4.

The calculation of the isosteric heat of adsorption involved the construction of a plot of ln(p) versus 1/T (Figure 5).

**FIGURE 6:**
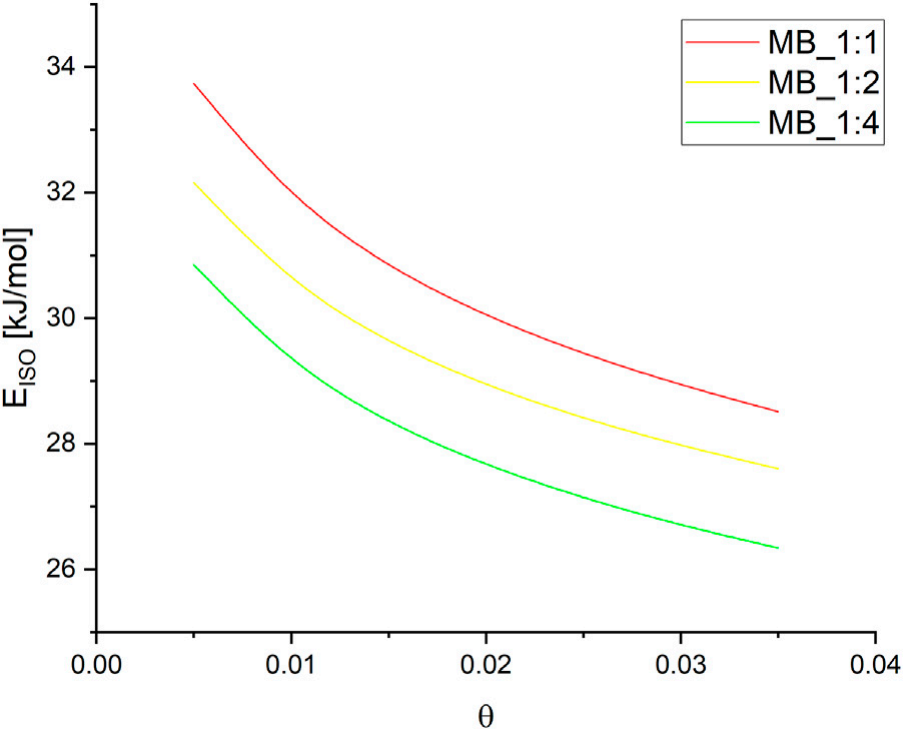
The isosteric heat of adsorption forMB_1:1, MB_1:2, MB_1:4.

The Sips Supplementary Equation S3 was used to calculate the pressures for a given surface coverage, and the resulting parameters are listed in Supplementary Table S2. Subsequently, the isosteric heat of adsorption was determined as a function of surface coverage for MB_1:1, MB_1:2, and MB_1:4, as shown in Figure 6.

The isosteric heat of adsorption exhibited a decreasing trend with increasing surface coverage of the activated carbons. The curves observed in the plot supported the physical nature of CO_2_ adsorption over all activated carbons. It was noted that for all samples, the isosteric heat of adsorption reduced rapidly. During the initial stage of adsorption, CO_2_ molecules penetrated the smallest micropores, leading to a strong interaction between the carbon surface and CO_2_, thus resulting in high isosteric heat at lower coverage. As CO_2_ adsorption increased, other pores were also involved, and the CO_2_-adsorbent surface interactions became weaker. CO_2_ molecules not only covered the surface but also filled the pore volume. A similar observation was also proposed by Abdulsalam et al. (2020). In Figure 5, the isosteric heat of adsorption varied from 34 to 28 kJ/mol for MB_1:1, 32 to 27 kJ/mol for MB_1:2, and 31 to 26 kJ/mol for MB_1:4. The values of the isosteric heat of adsorption were comparable to those presented in the literature, such as 31–25 kJ/mol (Zhou et al., 2022), approximately 38.9 kJ/mol (Rehman et al., 2022), and 28–18 kJ/mol (Sharma and Snyder, 2022).

**FIGURE 7:**
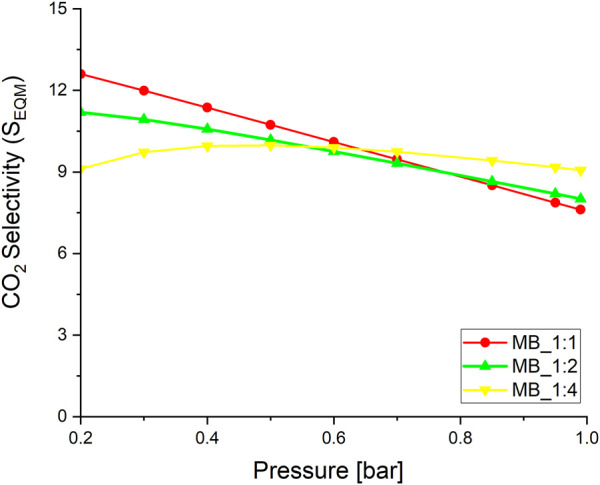
The CO_2_ over N_2_ selectivity, temperature 20°C.

Evaluating sorbents for CO_2_ removal from flue gas requires a clear definition of selectivity. To achieve this, measurements of nitrogen adsorption isotherms were conducted at 20°C and up to a pressure of 1 bar (refer to Supplementary Figure S4). The results indicated that N_2_ adsorption is directly proportional to the total pore volume. Therefore, the higher the total pore volume, the greater the N_2_ adsorption capacity. To calculate the selectivity ratio of CO_2_ over N_2_, the CO_2_ adsorption capacity was divided by the N_2_ adsorption capacity at 20°C (9) and presented at Figure 7:

$$S_{{CO}_{2}}=\frac{q_{{CO}_{2}\left(p\right)}}{q_{N_{2}\left(p\right)}}$$ (9)

where q_i(p)_ is the adsorption capacity [mmol/g] at the same partial pressure p; i is N_2_ or CO_2_, respectively.

An increase in pressure resulted in a gradual rise in the CO_2_/N_2_ selectivity ratio, except the activated carbon MB_1:4, whereas for the pressure up to 0.4 bar slow increase were observed. The best sorbent MB_1:4 exhibited a relatively high CO_2_/N_2_ selectivity ratio of 9.1 at 1 bar at 20°C. Activated carbons derived from Eucaliptus saw dust exhibited the CO_2_/N_2_ selectivity ratio equal to 5.4 at 1 bar at 20°C (Sevilla and Fuertes, 2011), petroleum pitch has 2.4 at 1 bar and 20°C (Wang and Liu, 2014), polyaniline 8. 4 at 1 bar and 20°C (Xie and Suh, 2013). Thus, it can be clearly seen that those adsorbents had lower selectivity for CO_2_ over N_2_ than those obtained and described in this paper. The experiments conducted in this study were carried out at a slightly lower temperature of 20°C. However, research on the selectivity of CO_2_ over N_2_ is limited, and most studies have been conducted at 20°C.

The study also employed the ideal adsorbed solution theory (IAST) to forecast the adsorption of CO2 and N2 mixtures using single gas adsorption isotherms of CO_2_ and N_2_ (10):

$$S_{IAST}=\frac{q_{{CO}_{2}}@p_{{CO}_{2}}/q_{N_{2}}@pN_{2}}{p_{N_{2}}/p_{{CO}_{2}}}$$ (10)

where qi@pi–adsorption capacity of i at the pressure pi

The IAST method can be utilized to determine the selectivity of CO_2_ over N_2_ under flue gas conditions, where the partial pressures of N_2_ and CO_2_ are 0.85 and 0.15, respectively. Supplementary Table S3 illustrates the selectivity calculated by the IAST method. The range of S_IAST_ values was from 13.2 to 16.6, which was comparable to the values reported for activated carbons derived from different biomass sources, e.g., for activated carbons derived from olive pomace S_IAST_ equals to 15.2 (Gonzalez et al., 2013), for activated carbons obtained from algae-glucose S_IAST_ is 17.3 (Sevilla et al., 2012). The most microporous activated carbon exhibited the highest value of the S_IAST_. Moreover, the S_IAST_ values decrease along with increase of the carbonaceous material:activating agent ratio.

## 4 Conclusion

Carbonaceous materials produced from the beet molasses during hydrothermal synthesis derived spherical structure. After activation by KOH, the morphology of those materials depends on the ratio of carbon material:KOH, i.e., the smaller amount of activator favors the spherical nature of obtained material.

The CO_2_ adsorption capacity of the activated carbons was evaluated at 0°C, 10°C, and 20°C. The results showed that the materials had a high specific surface area of up to 2005 m^2^g^−1^ and a total pore volume of up to 0.851 cm^3^g^−1^, with the MB_1:4 activated carbon exhibiting the highest values. At 0°C and 1 bar, the carbons were able to adsorb as much as 7.1 mmolg^−1^ of CO_2_. Although these findings are preliminary, they are promising, and further optimization of the synthesis parameters could lead to even higher adsorption capacities. The high microporosity of the activated carbons was found to be positively correlated with CO_2_ adsorption, and the isosteric heat of adsorption data suggested that the CO_2_ sorption mechanism was physical in nature. It is crucial to mathematically characterize CO_2_ adsorption to develop effective CO_2_ capture strategies.

## Data Availability

The original contributions presented in the study are included in the article/Supplementary Material, further inquiries can be directed to the corresponding author.
