# Supplementary material for: Activated biocarbons derived from molasses as new tailored CO2 adsorbents
Source: Front Chem. 2023 Jun 19;11:1184389. doi: 10.3389/fchem.2023.1184389 (PMC10315491; doi:10.3389/fchem.2023.1184389)
Supplement: Supplementary file 1 [file DataSheet1.docx]

Supplementary Material

Activated biocarbons derived from molasses as a new tailored CO_2_ adsorbents

Karolina Kiełbasa*

*** Correspondence:** Corresponding Author: karolina.kielbasa@zut.edu.pl

# Supplementary Data

**Langmuir Isotherm**

The Langmuir isotherm, which is the simplest model, was designed to characterize the adsorption of the gas-solid phase. It is also used to quantify and compare the maximum adsorption capacity of different sorbents. The Langmuir theory postulates monolayer coverage of adsorbate; adsorption occurs at specific homogeneous sites (all sites are equal, resulting in equal adsorption energies). Once an adsorbate molecule occupies a site, no more adsorption can take place at that site. The sorbent has a limited capacity for the adsorbate (Elmorsi, 2011; Günay et al., 2007):

$\boldsymbol{q=}\frac{\boldsymbol{q}_{\boldsymbol{mL}}\boldsymbol{b}_{\boldsymbol{L}}\boldsymbol{p}}{\boldsymbol{1+}\boldsymbol{b}_{\boldsymbol{L}}\boldsymbol{p}}$ **(S1)**

where q_mL_ is the maximum adsorption capacity [mmol/g], b_L_ is the Langmuir constant [bar^-1^], p is pressure [bar], q is the adsorbed quantity under p pressure [mmol/g].

**Freundlich Isotherm**

The Freundlich model describing an adsorption on heterogeneous surfaces with different adsorption energies (Ayawei et al., 2015a; Ayawei et al., 2015b) according to equation (S2):

$\boldsymbol{q=}\boldsymbol{k}_{\boldsymbol{F}}\boldsymbol{p}^{\boldsymbol{n}_{\boldsymbol{F}}}$ **(S2)**

where k_F_ is the Freundlich constant [mmol/g], n_F_ is the heterogeneity factor.

**Sips Isotherm**

The Sips model is frequently utilized for the description of the heterogeneous adsorbents such as for example, activated carbons (Delavar et al, 2010, Ning et al., 2006). It is reduced to the Freundlich model at low adsorbate concentrations, and at high adsorbate concentrations, it is similar to the Langmuir model (Travis and Etnier, 1981) and can be expressed by an equation (S3):

$\boldsymbol{q=}\frac{\boldsymbol{q}_{\boldsymbol{mS}}\boldsymbol{b}_{\boldsymbol{S}}\boldsymbol{p}^{\boldsymbol{n}_{\boldsymbol{S}}}}{\boldsymbol{1+}\boldsymbol{b}_{\boldsymbol{S}}\boldsymbol{p}^{\boldsymbol{n}_{\boldsymbol{S}}}}$ **(S3)**

where q_mS_ is the maximum adsorption capacity [mmol/g], b_S_ is the Sips constant [bar^-1^], n_S_ is the heterogeneity factor.

Analyzing the Sips model it should be noticed that the parameters presented in the Sips equation (S3) are dependent on the temperature:

$\boldsymbol{b}_{\boldsymbol{S}}\boldsymbol{=}\boldsymbol{b}_{\boldsymbol{0}}\boldsymbol{exp(}\frac{\boldsymbol{Q}}{\boldsymbol{R}\boldsymbol{T}_{\boldsymbol{0}}}\boldsymbol{)(}\frac{\boldsymbol{T}_{\boldsymbol{0}}}{\boldsymbol{T}}\boldsymbol{-1)}$ **(S4)**

$\boldsymbol{n}_{\boldsymbol{S}}\boldsymbol{=}\boldsymbol{n}_{\boldsymbol{0}}\boldsymbol{+\alpha(1-}\frac{\boldsymbol{T}_{\boldsymbol{0}}}{\boldsymbol{T}}\boldsymbol{)}$ **(S5)**

$\boldsymbol{qm}_{\boldsymbol{S}}\boldsymbol{=q}\boldsymbol{m}_{\boldsymbol{0}}\boldsymbol{exp}\left\{ \boldsymbol{x(1-}\frac{\boldsymbol{T}_{\boldsymbol{0}}}{\boldsymbol{T}}\boldsymbol{)} \right\}$ **(S6)**

**Toth Isotherm**

The Toth isotherm model is next empirical equation developed to enhance isotherm fitting between experimental and calculated data. The Toth isotherm model is useful in describing heterogeneous adsorption systems, which settles with both low and high-end boundaries of the concentration (Jafari Behbahani and Jafari Behbahani, 2014; Padder and Majunder, 2012) according to equation (S7):

$\boldsymbol{q=}\frac{\boldsymbol{q}_{\boldsymbol{mT}}\boldsymbol{b}_{\boldsymbol{T}}\boldsymbol{p}}{\boldsymbol{(1+(}\boldsymbol{b}_{\boldsymbol{T}}\boldsymbol{p}\boldsymbol{)}^{\boldsymbol{nT}}\boldsymbol{)}^{\frac{\boldsymbol{1}}{\boldsymbol{nT}}}}$ **(S7)**

where q_mT_ is the maximum adsorption capacity [mmol/g], b_T_ is the Toth constant [bar^-1^], n_T_ is the heterogeneity factor.

**Fritz-Schlunder Isotherm**

Fritz and Schlunder elaborated an empirical equation (S8) that can fit a wide range of experimental results because of many coefficients in the isotherm (Yaneva et al, 2013):

$\boldsymbol{q=}\frac{\boldsymbol{q}_{\boldsymbol{mFS}}\boldsymbol{b}_{\boldsymbol{FS}}\boldsymbol{p}}{\boldsymbol{1+}\boldsymbol{q}_{\boldsymbol{mFS}}\boldsymbol{p}\boldsymbol{n}_{\boldsymbol{FS}}}$ **(S8)**

where q_mFS_ is the maximum adsorption capacity [mmol/g], b_FS_ – the Fritz-Schlunder constant [bar^-1^], n_FS_ is the Fritz-Schlunder model exponent.

**Radke-Prausnitz Isotherm**

The Radke-Prausnitz model has several significant properties that make it the preferred choice for most adsorption systems with low adsorbate concentrations. At a low adsorbate concentration, the isotherm model reduces to a linear isotherm. At a high adsorbate concentration, it approaches the Freundlich isotherm, and when n_RP_ = 0, it becomes a Langmuir isotherm. Another essential property of this isotherm is that it gives a good fit over a wide range of adsorbate concentrations. Radke – Prausnitz equation can be expressed as (S9) (Radke and Prausnitz, 1972):

$\boldsymbol{q=}\frac{\boldsymbol{q}_{\boldsymbol{mRP}}\boldsymbol{b}_{\boldsymbol{RP}}\boldsymbol{p}}{\boldsymbol{(1+}\boldsymbol{b}_{\boldsymbol{RP}}\boldsymbol{p}\boldsymbol{)}^{\boldsymbol{n}_{\boldsymbol{RP}}}}\boldsymbol{,}$ **(S9)**

where q_mRP_ is the maximum adsorption capacity [mmol/g], b_RP_ is the Radke-Prausnitz constant [bar^-1^], n_RP_ is Radke-Prausnitz model exponent.

**The sum of the squares of the errors**

The sum of the squares of the errors (SSE) can be expressed as follows (S10) (Ho, 2004):

$\boldsymbol{SSE=}\sum_{\boldsymbol{i=1}}^{\boldsymbol{n}} \boldsymbol{(}{\boldsymbol{q}_{\boldsymbol{e,calc}}\boldsymbol{-}\boldsymbol{q}_{\boldsymbol{e,exp}}\boldsymbol{)}}_{\boldsymbol{i}}^{\boldsymbol{2}}$ **(S10)**

# Supplementary Figures and Tables

## Supplementary Figures

**Supplementary Figure S1.** The plots: ln(q_m_) versus T (a), ln(b) versus 1/T (b), and n versus 1/T (c) applied to the calculation of the Sips parameters in equations (S4-S6) for MB_1:1

**Supplementary Figure S2.** The plots: ln(q_m_) versus T (a), ln(b) versus 1/T (b), and n versus 1/T (c) applied to the calculation of the Sips parameters in equations (S4-S6) for MB_1:2

**Supplementary Figure S3.** The plots: ln(q_m_) versus T (a), ln(b) versus 1/T (b), and n versus 1/T (c) applied to the calculation of the Sips parameters in equations (S4-S6) for MB_1:4

**Supplementary Figure S4.** N_2_ adsorption at a temperature of 20 °C

## Supplementary Tables

Supplementary Table S1. Parameters of the Sips model for CO_2_ adsorption at different temperatures

|  | **MB_1:1** | | | **MB_1:2** | | | **MB_1:4** | | |
| --- | --- | --- | --- | --- | --- | --- | --- | --- | --- |
| Temperature | q_mS_ | b_S_ | n_S_ | q_mS_ | b_S_ | n_S_ | q_mS_ | b_S_ | n_S_ |
| [^o^C] | [mmol/g] | [bar^-1^] |  | [mmol/g] | [bar^-1^] |  | [mmol/g] | [bar^-1^] |  |
| 0 | 9.447 | 1.132 | 0.712 | 17.279 | 0.546 | 0.747 | 26.231 | 0.365 | 0.801 |
| 10 | 8.993 | 0.926 | 0.739 | 17.032 | 0.429 | 0.757 | 27.577 | 0.285 | 0.847 |
| 20 | 8.648 | 0.730 | 0.760 | 16.646 | 0.339 | 0.786 | 27.495 | 0.208 | 0.853 |

**Supplementary Table S2.** Parameters determined from Sips equation

| Activated Biocarbon | b_0_  [bar^-1^] | Q [kJ/mol] | n_0_ | α | χ | q_mo_ [mmol/g] |
| --- | --- | --- | --- | --- | --- | --- |
| **MB_1:1** | 0.66 | 15 | 0.76 | 0.59 | 1.27 | 9.42 |
| **MB_1:2** | 0.30 | 16 | 0.78 | 0.55 | 0.56 | 17.31 |
| **MB_1:4** | 0.18 | 19 | 0.86 | 0.65 | -0.59 | 26.49 |

**Supplementary Table S3.** CO_2_/N_2_ selectivity calculated on the basis of Ideal Adsorbed Solution Theory (IAST)

| Activated Biocarbon | S_IAST_ |
| --- | --- |
| **MB_1:1** | 16.5 |
| **MB_1:2** | 14.2 |
| **MB_1:4** | 13.2 |

**Supplementary References**

1. Elmorsi, T. M. (2011). Equilibrium isotherms and kinetic studies of removal of methylene blue dye by adsorption onto miswak leaves as a natural adsorbent. *J. Environ. Prot. Ecol.* 2:817–827.
2. Günay, A.; Arslankaya, E.; Tosun, I. (2007). Lead removal from aqueous solution by natural and pretreated clinoptilolite: adsorption equilibrium and kinetics. *J. Hazard. Mater.* 146:362–371.
3. Ayawei, N.; Angaye, S. S.; Wankasi, D.; Dikio, E. D. (2015). Synthesis, characterization and application of Mg/Al layered double hydroxide for the degradation of congo red in aqueous solution. *Open Journal of Physical Chemistry* 5: 56–70.
4. Ayawei, N.; Ekubo, A. T.; Wankasi, D.; Dikio, E. D. (2015). Adsorption of congo red by Ni/Al – CO_3_: equilibrium, thermodynamic and kinetic studies. *Oriental Journal of Chemistry* 31:1307–1318.
5. Delavar, M.; Ghoreyshi, A.A.; Jahanshahi, M.; Irannejad, M. (2010). Experimental evaluation of methane adsorption on granular activated carbon (GAC) and determination of model isotherm. *Engineering and Technology* 62:47−50.
6. Ning, P.; Li, F.; Yi, H.; Tang, X.; Peng, J.; Li, Y.; He, D.; Deng, H. (2012). Adsorption equilibrium of methane and carbon dioxide on microwave-activated carbon. *Sep. Purif. Technol.* 98:321−326.
7. Travis, C. C.; Etnier, E. L. (1981). A survey of sorption relationships for reactive solutes in soil. *J. Environ. Qual.* 10:8–17.
8. Jafari Behbahani, T.; Jafari Behbahani, Z. (2014). A new study on asphaltene adsorption in porous media. *Petroleum and Coal* 56:459–466.
9. Padder, M. S.; Majunder, C. B. C. (2012). Studies on Removal of As(II) and S(V) onto GAC/MnFe. *Compos. Interface.* 23:327–372.
10. Yaneva, Z. L.; Koumanova, B. K.; Georgieva, N. V. (2013). Linear regression and nonlinear regression methods for equilibrium modelling of p – nitrophenol biosorption by Rhyzopus oryzen: comparison of error analysic criteria. *J. Chem.* NY517631.
11. Radke, C.J.; Prausnitz, J.M. (1972). Adsorption of organic solutions from dilute aqueous solution on activated carbon. *Ind. Eng. Chem. Fundam. 11*:445–451.
12. Ho, Y.S. (2004). Selection of optimum sorption isotherm. *Carbon* 42:2115–2116.
